# Early maladaptive schemas as predictors of maternal bonding to the unborn child

**DOI:** 10.1186/s40359-019-0297-9

**Published:** 2019-04-11

**Authors:** Dag Nordahl, Ragnhild Sørensen Høifødt, Agnes Bohne, Inger Pauline Landsem, Catharina Elisabeth Arfwedson Wang, Jens C. Thimm

**Affiliations:** 1 Department of Psychology, Faculty of Health Sciences, UiT The Arctic University of Norway, 9037 Tromsø, Norway; 2 Division of Child and Adolescent Health, University Hospital of North Norway, 9038 Tromsø, Norway; 3 Division of Mental Health and Addiction, University Hospital of North Norway, 9016 Tromsø, Norway; 4 Department of Health and Care Sciences, Faculty of Health Sciences, UiT The Arctic University of Norway, 9037 Tromsø, Norway

**Keywords:** Early maladaptive schemas, Maternal cognitions, Mediation, Maternal-fetal bonding, Maternal-fetal attachment, Antenatal depression

## Abstract

**Background:**

The quality of an expectant mother’s bonding to the fetus has been shown to be associated with important developmental outcomes. Previous studies suggest that bonding quality is predicted by, for example, social support, psychological well-being, and depression. However, little is known regarding the role of maternal cognition in maternal-fetal bonding. Early maladaptive schemas (EMSs) are negative and stable assumptions about oneself and one’s relationships with others that are developed during childhood and adolescence. In the present study, we examined the associations between EMSs and the quality of the bonding to the fetus in expectant mothers.

**Methods:**

The present investigation is part of a larger study in which 220 pregnant women (approximately 12% of the pregnant women in the region) and 130 of their partners were recruited from October 2015 until December 2017. The sample for the current study comprised 165 pregnant women (mean age 30.8 years, SD 4.1 years). The participants completed the Young Schema Questionnaire Short Form 3 (YSQ-S3) between gestational weeks 24 and 37 and the Maternal Antenatal Attachment Scale (MAAS) and the Edinburgh Postnatal Depression Scale (EPDS) between gestational weeks 31 and 41.

**Results:**

All EMS domains correlated significantly and negatively with scores for quality of maternal-fetal bonding on the MAAS. Only the Disconnection and Rejection domain correlated significantly and negatively with MAAS scores for intensity of preoccupation with the fetus. The Disconnection and Rejection domain was a significant independent predictor of the quality of maternal-fetal bonding. Symptoms of depression mediated the effect of the EMS domains on the quality of maternal-fetal bonding. The EMS domains Disconnection and Rejection, Impaired Autonomy and Performance, and Impaired Limits showed significant direct effects on bonding quality.

**Conclusions:**

EMSs are related to expectant mothers’ self-reported bonding to their fetuses. This association was mediated by the mothers’ symptoms of depression. The results may have implications for the early identification of pregnant women at risk of bonding difficulties and encourage more studies on cognitive schemas and mechanisms for maternal-fetal bonding.

## Background

### Introduction

Maternal bonding is described as an emotional tie or bond from a mother towards her child [1]. Maternal bonding starts developing during pregnancy [2], and this development continues after birth [3–7]. Bonding is clearly related to the concept of attachment, and these two terms are sometimes used interchangeably. However, there is an important distinction between bonding and attachment. In Bowlby’s attachment theory [8], the attachment system has the purpose of eliciting caregiving behavior from important others, which pregnant women do not seek from their fetus. Hence, some have argued that attachment is an inappropriate term for a mother’s emotional tie to her fetus [9, 10] and use other labels, such as bonding. One way to measure bonding during pregnancy is through the mother’s descriptions of the qualities of the affective experience towards the fetus, thoughts about the fetus and reactions to experiences of loss. Additionally, one can measure the mother’s intensity of preoccupation with the fetus [1]. Bonding as early as during pregnancy has been shown to be related to a variety of infant outcomes, including colic, infant temperament difficulties, and delayed developmental milestones [11]. In addition, maternal-fetal bonding predicts the quality of mother-infant interaction after birth [12, 13], which has been shown to be important for the child’s development [14–17]. Therefore, research on factors that contribute to explaining different qualities of maternal bonding is warranted. Knowledge of predictors of maternal bonding during pregnancy may aid in the development of interventions to enhance bonding for at-risk mothers before the child is born, interaction difficulties become established and developmental difficulties are manifested.

A number of predictors of maternal-fetal bonding have been examined and reviewed by Cannella [18] and Alhusen [19]. However, the findings were inconsistent for most variables. Among variables with some findings of positive relationships with maternal-fetal bonding were social support [18], family support [19], psychological well-being, having an ultrasound test performed [19], and attitude towards childbearing [18]. Variables with some indications of a negative relationship with maternal-fetal bonding included substance abuse [19], anxiety [19], maternal age [18], being married [18], and experience with motherhood [18]. Other studies have shown a positive association between the quality of the relationship with one’s own mother in childhood and bonding to the fetus during pregnancy [20, 21]. In addition, pregnant women’s attachment style in romantic relationships relates to maternal-fetal bonding [22–24]. For example, securely attached women reported a higher quality of maternal-fetal bonding than insecurely attached women [24]. In line with the findings on adult attachment styles, personality traits in the mother such as agreeableness, extroversion and conscientiousness also relate positively to maternal-fetal bonding during pregnancy [2]. It has also been observed that mothers’ level of rumination predicts maternal-fetal bonding [25]. Another important factor for maternal bonding is maternal depression. Depressed mood is prevalent in pregnancy, affecting approximately 10% of pregnant women [26]. A meta-analysis from 2009 found that depressive symptoms have a small effect on maternal-fetal bonding [27]. However, more recent studies have shown that maternal depressive symptoms in early pregnancy have a negative impact on maternal-fetal bonding in late pregnancy [28]. In two studies, one of first-time mothers and the other of low-income women, depressive symptoms were associated with the quality of maternal feelings towards the fetus [29, 30]. Furthermore, pregnant women with clinical depression have been shown to have reduced levels of maternal-fetal bonding compared to those without depression [31]. Despite the variety of potential predictors of maternal-fetal bonding that have been investigated, there has been a paucity of studies on the role of the mother’s cognitions about herself and her relationship with others regarding her bonding to the fetus.

### Early maladaptive schemas, attachment theory, and bonding

The present study explored how mothers’ early maladaptive schemas (EMSs), which can be described as negative emotional and cognitive patterns regarding oneself and one’s relationships with others [32], are associated with bonding towards the fetus. According to Young et al. [32], EMSs develop during childhood and adolescence from an interplay between the child’s temperament and adverse experiences with parents and peers. The theory states that EMSs result from unmet core emotional needs in the areas of secure attachments, independence, competence, sense of identity, autonomy to express needs and emotions, naturalness and play, and reasonable limits and self-mastery [32]. EMSs are elaborated throughout life, are dysfunctional, and guide the view of one self and one’s relationship with others [32]. The most recent list of EMSs includes 18 EMSs organized into four EMS domains according to a recent revision [33] (see Table 1). Individuals with EMSs from the domain of 1) Disconnection and Rejection expect that their needs for secure attachments, social belonging, nurturance, love, and spontaneity will not be consistently met. People who score high on the EMS domain 2) Impaired Autonomy and Performance have negative assumptions about their own capability to function independently in daily life and inadequacy in regard to areas of achievement. The EMS domain 3) Excessive Responsibility and Standards involves a strong focus on following rigid internalized rules and expectations with regard to many aspects of life, such as obligations, good behavior and orderliness, at the expense of one’s own well-being, health, or interpersonal relationships. Individuals high in this domain may also feel egoistic or guilty if they occupy themselves with positive activities. Finally, the EMS domain 4) Impaired Limits refers to difficulties in self-directed behavior towards goal achievement, lack of frustration tolerance, and deficits in internal limits, which may be manifested as feelings of superiority or feelings of being entitled to privileges [34].

**Table 1:** Short descriptions of the 18 early maladaptive schemas proposed by Young et al. [32] and their organization into four schema domains [33]

Schema domains and early maladaptive schemas	Short descriptions
Disconnection and Rejection domain	
Emotional deprivation	The assumption that others will not meet one’s emotional needs.
Social isolation	A sense that one is set apart/different from other people.
Emotional inhibition	The tendency to suppress the expression of emotions and to have difficulties relating freely to others.
Defectiveness/shame	The assumption that one is full of flaws, and if these are exposed, one would lose the respect or love of others.
Mistrust	Distrust in others’ intentions or expected abuse.
Negativity/pessimism	The inclination to focus on the negative areas in life, with an expectation that things will end badly.
Impaired Autonomy and Performance domain	
Dependence/incompetence	The assumption that one is incapable of handling everyday obligations without substantial assistance from others.
Failure to achieve	A conviction that one is a failure in regard to achievements.
Subjugation	Surrender of control to other people, due to the fear of negative reactions, usually implying a belief that one’s thoughts and feelings are not important.
Abandonment	A feeling of instability in support from significant others.
Enmeshment	Over involvement with significant others.
Vulnerability to harm	Fear of medical, mental and/or external catastrophes.
Excessive Responsibility and Standards domain	
Self-sacrifice	The tendency to prioritize others’ needs ahead of one own needs.
Unrelenting standards	The assumption that one must meet one’s own high standards of achievement and behavior.
Punitiveness	The assumption that one and others should be disciplined for mistakes.
Impaired Limits domain	
Entitlement	A conviction of superiority.
Approval-seeking	The tendency to seek approval and connection with other people and to be sensitive to the reactions of other.
Insufficient self-control	Challenges with frustration tolerance and self-control.

Essentially, EMSs resemble the internal working model in attachment theory [35], as both are assumed to develop during childhood from interpersonal experiences with important others and to have a complex influence on how one relates to oneself and to other people [32]. For example, the quality of parental relations and rearing in childhood has been found to be associated with EMSs [36–39] and attachment style [40, 41] in adolescence and adulthood. Moreover, insecure attachment early in life has been shown to be related to increased signs of EMSs 15 years later [42], and attachment style in adulthood is found to be related to EMSs [43]. EMSs are suggested to mediate between adverse childhood experiences with parents and adult interpersonal functioning [36]; they relate to interpersonal problems [44], and they may also play a role in parent-infant relationships, such as infant feeding difficulties [45, 46]. Hence, EMSs are related to attachment theory, are tightly intertwined with social functioning and relationships, and may therefore contribute to the understanding of the mechanisms underlying maternal-fetal bonding.

Finally, EMSs are assumed to play a central role in the development of later psychopathology [32]. Several EMSs have been found to be related to depressive symptom severity [47–52]. This includes defectiveness/shame, failure, and self-sacrifice [48] as well as defectiveness/shame, insufficient self-control, vulnerability, and incompetence/inferiority [49]. As depression is associated with weakened bonding, it is conceivable that the relationship between EMS and bonding is mediated by depressive symptoms.

### Objectives and aims of the present study

The main objective of the present study is to examine the relationship between mothers’ EMSs and two aspects of maternal-fetal bonding: the intensity of preoccupation with the fetus and the quality of the affective bond [1]. The EMS domain Disconnection and Rejection is theorized to impact the development of close relationships [32]. Hence, we hypothesize that this domain in particular will relate negatively to maternal bonding. Furthermore, as EMSs can be seen as emotional and cognitive scripts impacting experiences of oneself and one’s relationships [32], we hypothesize that EMS domains relate more to the qualitative experiences of bonding (quality) than to the amount of time mothers are consumed with thinking or talking about the fetus (intensity of preoccupation) [1]. Previous research [47] has demonstrated that a) EMSs are related to depression and b) that depression predicts maternal bonding [27, 28]. Accordingly, we also sought to explore whether the EMS domains have a direct effect on maternal bonding or whether this relationship is mediated by depressive symptoms.

## Method

### Participants and procedure

The present study is part of the Northern Babies longitudinal study on parental and infant prenatal risk factors, parent-infant interaction and infant development [53]. All Norwegian-speaking pregnant women and partners thereof who lived in the municipality of Tromsø were eligible for inclusion. The recruitment period lasted from October 2015 until December 2017. Participants were recruited by midwives who informed pregnant women and their families about the study. Potential participants who agreed to be contacted were later telephoned by a member of the research team for more information about the study and to plan a meeting for inclusion in the study. In this phone call, the researcher encouraged the participation of both parents, and partners were invited to the meeting for further information about the study and inclusion. A total of 430 pregnant women agreed to be contacted by phone. Two hundred and twenty pregnant women (equivalent to approximately 12% of the pregnant women in the region) and 130 partners consented to be included in the study. The reasons for exclusion included failure to respond to the phone call and refusal to participate in the study due to time considerations. The families were followed longitudinally at six measurement points (T1-T6), including three time points during pregnancy (T1-T3) and three postpartum until the infant was 6 months old (T4-T6).

In the present study, all pregnant women who had completed measures of EMSs and bonding to the fetus (administered at T2 and T3, respectively) were included (*n* = 165). Reasons for exclusion were omission of the T2 measurement due to late inclusion (*n* = 13), omission of the T3 measurement due to closeness in time to T2 (*n* = 1), withdrawing from the study or not answering all or relevant parts of T2 or T3 (*n* = 30), answering T2 and T3 successively on the same day (*n* = 3), premature birth (*n* = 3), and answering T3 after giving birth (*n* = 3). Furthermore, data from two participants could not be identified and were excluded from the sample. T1 ranged from gestational week 13 to week 30 (mean week 22.3). T2 measures were administered between gestational weeks 24 and 37 (mean week 28.3). T3 measures were administered between gestational weeks 31 to 41 (mean week 34.2). The overlap in timing between the steps in this study is largely due to variations in gestational week at inclusion (T1) and late responses to later steps for some participants. The time between T1 and T2 ranged from 1 to 17 weeks (mean 6 weeks, SD 2.14). The time between T2 and T3 ranged from 1 to 13 weeks (mean 5.9 weeks, SD 2.14). At T2 and T3, participants completed questionnaires using an online survey tool. Further details about the design and procedure have been published previously [53].

### Measures

Demographic information was collected at T1 and included questions about maternal age, whether pregnancy was wanted, number of children, education, income and marital status, as well as questions about previous mental health status and help sought for mental health issues. In addition, at T3, participants answered a question about having undergone ultrasound tests during their current pregnancy.

EMSs were measured using the Young Schema Questionnaire Short Form 3 (YSQ-S3; [54]) at T2. The YSQ-S3 is a self-reported measure consisting of 90 items. The items are rated on a 6-point Likert scale ranging from [1] “Completely untrue of me” to [6] “Describes me perfectly”. The 18 EMSs constituting the YSQ-S3, their organization into four domains according to recent research [33] and short descriptions of the schemas are shown in Table 1. We used the following four domains in the present study: Disconnection and Rejection (30 items), Impaired Autonomy and Performance (30 items), Excessive Responsibility and Standards (15 items), and Impaired Limits (15 items). The present Norwegian version of the YSQ-S3 has been used in earlier research [55]. In the present study, the four domains of the YSQ-S3 had adequate internal consistency (see Table 3).

Bonding felt by the mother towards her baby during pregnancy was measured with the Maternal Antenatal Attachment Scale (MAAS; [1]) at T3. This self-report measure consists of 19 statements. The statements are followed by individual response options rated on 5-point Likert scales, for example, ranging from “Very emotionally distant from my baby” to “Very close emotionally to my baby”. Higher values indicate higher bonding. In addition to a global scale (19 items), the measure consists of two subscales: [1] quality of maternal bonding (QMB; 10 items) and [2] intensity of preoccupation with the fetus (IPF; 8 items). Following guidelines from the author of the scale, one item was excluded from the subscales [56]. QMB assesses emotions towards the unborn child. The time spent in bonding mode with the unborn child is measured with the IPF. The present study focuses on the two subscales. Members of the research group translated the original version of MAAS to Norwegian, and a professional translator checked the translation and provided suggestions for improvement. In the present sample, the two subscales had adequate internal consistency (see Table 3).

Maternal symptoms of depression were measured with the Edinburgh Postnatal Depression Scale (EPDS; [57]) at T3. The EPDS is a self-report inventory consisting of 10 items and used as a screening instrument for depression during pregnancy and after birth [58]. The EPDS includes items concerning sadness, anxiety, sleep and thoughts of harming oneself. Each item is scored on a 4-point scale with individual response options across items. The maximum score is 30. Higher scores indicate more symptoms of depression, and the cut-off for probable clinical depression is a score of 13 or more [57, 59]. The current study applied the measure as a continuous scale. The Norwegian translation of the EPDS has been used in previous research [60]. In the present sample, EPDS had adequate internal consistency (see Table 3).

### Approach to data analysis and missing data

Skewness and/or kurtosis were above 1 for all scales except MAAS IPF and the YSQ-S3 domain Impaired Limits. As this indicates non-normal distributions, nonparametric approaches were used. Spearman correlations were conducted, and for regression and mediation analysis, a bootstrapping percentile approach with 10,000 samples was used to generate confidence intervals. Hierarchical regression analyses were employed to test whether the four EMS domains predicted maternal bonding. In block one, we controlled for seven potentially confounding variables (e.g., maternal age, education, parenting experience and mental health history). These variables are listed in Table 2. The variables of maternal education and gross annual household income were dummy coded. Parenting experience was recoded to indicate first-time mothers and those with one or more previous children. The variables of marital status and wanting the pregnancy contained little variability in scores and were therefore excluded from the analysis. The four EMS domains were added in block two. Mediation analysis was carried out to explore whether symptoms of depression mediated the relationship between EMS domains and maternal bonding, controlled for potential confounding variables. Only significant EMS domains in the correlation analysis between EMS domains and bonding were tested in the mediation analysis.

**Table 2:** Sample demographics

Characteristics at T1	Mean (SD)	N (%)
Maternal age (years)^a^	30.8 (4.10)	
Pregnancy wanted^b^		
Yes		156 (94.5)
No		2 (1.2)
Do not know		3 (1.8)
Parenting experience		
First-time mother		84 (50.9)
Second-time mother		68 (41.2)
Two or more previous children		13 (7.9)
Maternal education		
Upper secondary school or lower		22 (13.3)
Up to 4 years of higher education		50 (30.3)
4 or more years of higher education		93 (56.4)
Gross annual household income		
350,000 NOK (44,980 USD) or less		6 (3.6)
351,000–750,000 NOK (45108–96,386 USD)		46 (27.9)
751,000 NOK (96,515 USD) or more		113 (68.5)
Marital status		
Married or cohabiting		162 (98.1)
Single		3 (1.8)
Maternal mental health history		
History of contact with professionals for mental health issues		50 (30.3)
Previous experience with being depressed most of the day, almost every day for a period of 2 weeks		55 (33.3)
History of a diminished ability to enjoy things one has usually found enjoyable for a period of 2 weeks		70 (42.4)

*N* = 161–165

^a^one missing value

^b^four missing values

Descriptive statistics, correlations and regression analysis were conducted with SPSS 25, and PROCESS version 3.0 [61] was used for mediation analysis.

To compute scale scores, we required more than 80% of the values to be present. No values were missing from the YSQ-S3 or the EPDS. Only 0.3% of the values from the MAAS QMB and IPF were missing. We decided not to replace the missing values.

## Results

Table 2 reports the demographic data. The mean age for the sample was 30.8 years. A large proportion of the women reported wanting the pregnancy (94.5%), currently living with a partner (98.1%) and having a gross annual household income above 751,000 NOK (96,515 USD) (68.5%). In addition, 162 (98.2%) participants (missing: *n* = 3) reported having at least one ultrasound test performed during the current pregnancy. Approximately half of the participants in the sample were first-time mothers (50.9%) and had four or more years of higher education (56.4%). A substantial number of participants reported that they had previously been depressed (33.3%) or were a diminished ability to enjoy things they usually find enjoyable (42.4%) for a period of 2 weeks. Furthermore, 30.3% had been in contact with professionals for mental health issues at some point during their life.

Table 3 reports the means, standard deviations and correlations for the study variables. With regard to maternal bonding, only MAAS QMB was significantly related (*p* < .001) to the total EPDS score, with a correlation of −.38. All EMS domains were significantly related (*p* < .001) to MAAS QMB. These correlations were negative and ranged from −.26 (Impaired Limits) to −.39 (Disconnection and Rejection). Only the EMS domain Disconnection and Rejection was significantly related (*p* < .05) to MAAS IPF (*r*_*s*_ = −.17). All EMS domains were significantly related (*p* < .001) to the total EPDS score. These correlations were positive and ranged from .37 (Impaired Limits) to .50 (Impaired Autonomy and Performance).

**Table 3:** Descriptive statistics, Cronbach’s alpha and Spearman correlations between the study measures

	Cronbach’s alpha	Mean	SD	Correlation		
				MAAS QMB	MAAS IPF	EPDS
YSQ-S3 DR	.94	1.62	0.56	−.39***	−.17*	.48***
YSQ-S3 IAP	.92	1.49	0.45	−.36***	−.11	.50***
YSQ-S3 ERS	.87	2.54	0.67	−.28**	−.09	.43***
YSQ-S3 IL	.83	1.99	0.52	−.26**	−.02	.37***
MAAS QMB	.79	44.94	4.13	–	.54***	−.38***
MAAS IPF	.77	27.15	4.69		–	−.13
EPDS	.88	4.19	4.33			–

*N* = 165; *YSQ-S3* Young Schema Questionnaire-Short Form 3, *DR* Disconnection and Rejection, *IAP* Impaired Autonomy and Performance, *ERS* Excessive Responsibility and Standards, *IL* Impaired Limits, *MAAS* Maternal Antenatal Attachment Scale, *QMB* quality of maternal bonding, *IPF* intensity of preoccupation with the fetus, *EPDS* Edinburgh Postnatal Depression Scale; *p < .05, **p < .001, ***p < .0001

Table 4 presents the results of the regression model with the MAAS QMB as the outcome. Prior to the regression analysis, indices of possible multicollinearity were examined due to high intercorrelations between the EMS domains (*r*_*s*_ = .58–.82). Variance inflation factors (1.97–4.90) and tolerance (0.20–0.51) indicate possible multicollinearity, although not at a level that would raise serious concern [62–64]. In the first block, potentially confounding variables were included as predictors. The regression model with only the confounding variables was significant (*p* = .002), explaining 15% of the variance of the MAAS QMB subscale. In the second block, the four EMS domains were included as predictors. The regression model was significant (*p* < .001), explaining 32% of the variance of the MAAS QMB subscale. The increase in explained variance from model one to model two was significant (*p* < .001). The EMS domain Disconnection and Rejection was a significant individual predictor (*p* = .045).

**Table 4:** Hierarchical regression analysis testing EMS domains as predictors of MAAS QMB

Block	Predictors	*b* (95% CI)	SE B	*β*	*p*	*R* ^*2*^
1	Maternal age	0.11 (− 0.06, 0.29)	0.09	.11	.230	.15
	Parenting experience	0.03 (− 1.25, 1.27)	0.64	.00	.958	
	Maternal education^a^					
	Upper secondary school or lower	1.98 (−0.40, 4.43)	1.23	.16	.105	
	Up to 4 years of higher education	1.07 (−0.46, 2.55)	0.77	.12	.167	
	Gross annual household income^b^					
	351,000–750,000 (45,108–96,386 USD)	−0.76 (−4.25, 3.01)	1.83	−.08	.663	
	750,000 or more (96,515 USD or more)	0.28 (− 2.89, 3.92)	1.72	.03	.866	
	Mental health help seeking	0.48 (− 1.37, 2.16)	0.90	.05	.598	
	Previous experience with being depressed	−2.11 (−3.88, −0.38)	0.89	−.24	.020	
	Previous lack of joy	−1.56 (−3.18, −0.08)	0.79	−.19	.052	
2	Maternal age	0.12 (−0.06, 0.29)	0.09	.12	.193	.32
	Parenting experience	−0.29 (−1.48, 0.86)	0.59	−.04	.625	
	Maternal education^a^					
	Upper secondary school or lower	1.59 (−0.45, 3.53)	1.02	.13	.123	
	Up to 4 years of higher education	0.96 (−0.50, 2.32)	0.72	.11	.192	
	Gross annual household income^b^					
	351,000–750,000 (45,108–96,386 USD)	−1.08 (−4.95, 2.96)	2.00	−.12	.571	
	750,000 or more (96,515 USD or more)	−0.49 (− 4.18, 3.44)	1.93	−.06	.785	
	Mental health help seeking	1.12 (−0.47, 2.67)	0.79	.12	.163	
	Previous experience with being depressed	−1.55 (−3.19, 0.03)	0.81	−.18	.063	
	Previous lack of joy	−0.89 (−2.49, 0.64)	0.79	−.11	.260	
	Disconnection and Rejection	−2.66 (−5.34, −0.23)	1.29	−.36	.045	
	Impaired Autonomy and Performance	−0.71 (−3.52, 1.70)	1.33	−.08	.593	
	Excessive Responsibility and Standards	0.67 (−0.79, 2.24)	0.76	.11	.386	
	Impaired Limits	−1.12 (−2.97, 0.54)	0.89	−.14	.213	

*EMS* early maladaptive schemas, *MAAS* Maternal Antenatal Attachment Scale, *QMB* quality of maternal bonding, *IPF* intensity of preoccupation with the fetus; ^a^variables were dummy coded with four or more years of higher education as a reference; ^b^variables were dummy coded with 350,000 NOK (44,980 USD) or less as reference; “Mental health help seeking” = having been in contact with professionals for mental health issues; “Previous experience with being depressed” = Previous experience with being depressed most of the day, almost every day for a period of 2 weeks; “Previous lack of joy” = Having previously had a 2-week period of diminished ability to enjoy things one has usually found enjoyable; confidence intervals and standard errors were based on 9986 bootstrap samples, as SPSS did not manage to generate the requested 10,000 samples; *N* = 163

Table 5 presents the result of the regression model with the MAAS IPF as the outcome. The first block with the potentially confounding variables as predictors was significant (*p* < .001), explaining 21% of the variance of the MAAS IPF subscale. In the second block, the four EMS domains were included as predictors. The regression model was significant (*p* < .001), explaining 25% of the variance of the MAAS IPF subscale. The increase in explained variance from model one to model two was not significant (*p* = .117). No EMS domain was significant as an individual predictor.

**Table 5:** Hierarchical regression analysis testing EMS domains as predictors of MAAS IPF

Block	Predictors	*b* (95% CI)	*SE B*	*β*	*p*	*R* ^*2*^
1	Maternal age	−0.24 (− 0.44, − 0.04)	0.10	−.21	.019	.21
	Parenting experience	−2.65 (−4.02, − 1.32)	0.69	−.28	.001	
	Maternal education^a^					
	Upper secondary school or lower	1.19 (− 1.22, 3.66)	1.24	.09	.333	
	Up to 4 years of higher education	0.69 (−0.87, 2.17)	0.77	.07	.372	
	Gross annual household income^b^					
	351,000–750,000 (45,108–96,386 USD)	2.48 (−2.88, 9.04)	3.05	.24	.379.	
	750,000 or more (96,515 USD or more)	3.67 (−1.72, 10.26)	3.04	.36	.187	
	Mental health help seeking	0.63 (−1.44, 2.46)	1.00	.06	.532	
	Previous experience with being depressed	−1.32 (−3.26, 0.44)	0.94	−.13	.162	
	Previous lack of joy	−0.92 (−2.78, 0.89)	0.93	−.10	.323	
2	Maternal age	−0.24 (− 0.44, − 0.03)	0.10	−.21	.026	.25
	Parenting experience	−2.75 (−4.11, −1.43)	0.68	−.29	.000	
	Maternal education^a^					
	Upper secondary school or lower	1.13 (−1.14, 3.41)	1.16	.08	.319	
	Up to 4 years of higher education	0.66 (−0.94, 2.18)	0.79	.06	.406	
	Gross annual household income^b^					
	351,000–750,000 (45,108–96,386 USD)	2.54 (−2.76, 9.13)	3.05	.24	.379	
	750,000 or more (96,515 USD or more)	3.55 (−1.85, 10.17)	3.07	.35	.210	
	Mental health help seeking	0.79 (−1.21, 2.61)	0.97	.08	.423	
	Previous experience with being depressed	−1.01 (−2.93, 0.76)	0.94	−.10	.276	
	Previous lack of joy	−0.54 (−2.29, 1.21)	0.90	−.06	.550	
	Disconnection and Rejection	−2.02 (−4.81, 0.62)	1.37	−.24	.143	
	Impaired Autonomy and Performance	0.46 (−3.11, 3.63)	1.71	.04	.785	
	Excessive Responsibility and Standards	−0.02 (−1.69, 1.67)	0.86	.00	.980	
	Impaired Limits	0.00 (−1.84, 1.83)	0.93	.00	.997	

*EMS* early maladaptive schemas, *MAAS* Maternal Antenatal Attachment Scale, *QMB* quality of maternal bonding, *IPF* intensity of preoccupation with the fetus; ^a^variables were dummy coded with four or more years of higher education as a reference; ^b^variables were dummy coded with 350,000 NOK (44,980 USD) or less as a reference; “Mental health help seeking” = having been in contact with professionals for mental health issues; “Previous experience with being depressed” = Previous experience with being depressed most of the day, almost every day for a period of 2 weeks; “Previous lack of joy” = Having previously had a 2-week period of diminished ability to enjoy things one has usually found enjoyable; confidence intervals and standard errors were based on 9979 bootstrap samples, as SPSS did not manage to generate the requested 10,000 samples; *N* = 163

Mediation analysis testing depressive symptoms as a mediator between EMS domains and maternal bonding was performed only for the EMS domains that correlated significantly with MAAS QMB and MAAS IPF scores. All potentially confounding variables from the hierarchical regression analysis were included as covariates in the mediation analysis. Confidence intervals for the direct and indirect effects were based on 10,000 bootstrap samples generated in PROCESS. Confidence intervals for the total effects were based on approximately 10,000 bootstrap samples generated in a series of regression analyses in SPSS. One participant was missing a value on the covariate maternal age, and the analyses are based on data from 164 participants. There were significant total effects of the EMS domains Disconnection and Rejection, *b* = − 3.150, 95% CI [− 4.898, − 1.482]; Impaired Autonomy and Performance, *b* = − 3.749, 95% CI [− 5.858, − 1.802]; Excessive Responsibility and Standards, *b* = − 1.471, 95% CI [− 2.496, − 0.357]; and Impaired Limits, *b* = − 2.426, 95% CI [− 4.022, − 0.951] on MAAS QMB. There was a significant direct effect of Disconnection and Rejection, *b* = − 1.789, 95% CI [− 3.315, − 0.523]; Impaired Autonomy and Performance, *b* = − 1.776, 95% CI [− 3.344, − 0.251]; and Impaired Limits, *b* = − 1.142, 95% CI [− 2.354, − 0.061] on the MAAS QMB subscale. There was no significant direct effect of Excessive Responsibility and Standards, *b* = − 0.333, 95% CI [− 1.337, 0.637], on the MAAS QMB. The EMS domains Disconnection and Rejection, *b* = − 1.361, 95% CI [− 2.503, − 0.448]; Impaired Autonomy and Performance, *b* = − 1.974, 95% CI [− 3.607, − 0.673]; Excessive Responsibility and Standards, *b* = − 1.138, 95% CI [− 2.009, − 0.404]; and Impaired Limits, *b* = − 1.285, 95% CI [− 2.471, − 0.392] had significant indirect effects on MAAS QMB scores through EPDS scores. The EMS domain Disconnection and Rejection showed a significant total effect on MAAS IPF scores, *b* = − 1.749, 95%, CI [− 3.320, − 0.292]. The EMS domain Disconnection and Rejection did not show a significant direct effect on MAAS IPF scores, *b* = − 1.146, 95%, CI [− 2.868, 0.275], or a significant indirect effect on MAAS IPF scores through EPDS scores, *b* = − 0.603, 95% CI [− 1.535, 0.201].

## Discussion

Earlier research has revealed a range of predictors of maternal-fetal bonding [18, 19], illustrating the complexity in explaining different qualities of bonding. Few studies have included cognitions [25], and no study so far has examined the role of mothers’ cognitive schemas regarding herself and her relationships with others. These schemas are thought to have roots in the mothers’ own relationship experiences with important others in childhood [32, 36–39] and are linked to attachment style [42, 43]. Thus, by focusing on EMSs, our findings may contribute to an enhanced understanding of the mechanisms underlying maternal bonding.

The present study investigated the relationship between mothers’ EMSs and the quality of maternal-fetal bonding as measured with MAAS. To the best of our knowledge, the present study is the first to explore these associations. Furthermore, as symptoms of depression are related to EMSs outside of pregnancy [47–50] and to maternal-fetal bonding [27–30], we also explored the mediating effects of symptoms of depression between EMS domains and maternal-fetal bonding. Our explorations revealed that all four EMS domains correlated significantly with bonding quality. Regression analyses showed that the four EMS domains and seven potentially confounding variables (e.g., maternal age, education, parenting experience and mental health history) explained a substantial part of the variance of bonding quality (32%). The EMS domain Disconnection and Rejection predicted the quality of maternal bonding above and beyond the other EMS domains. This finding supports our hypothesis that especially the EMS domain Disconnection and Rejection relates to maternal bonding. Mediation analyses revealed that the relations between all EMS domains and quality of bonding were mediated by symptoms of depression. Additionally, all EMS domains except Excessive Responsibility and Standards showed significant direct effects on bonding quality. This means that the domains had unique contributions to bonding quality when we controlled for symptoms of depression and the seven potentially confounding variables.

In line with our hypothesis, we found only a few links between EMS domains and the MAAS subscale measuring intensity of preoccupation with the fetus. Only the EMS domain Disconnection and Rejection correlated significantly with intensity of preoccupation, and no EMS domains emerged as significant unique predictors of scores on this subscale in the hierarchical regression model. Additionally, symptoms of depression did not emerge as a mediator between the EMS domain Disconnection and Rejection and intensity of preoccupation. In line with earlier research [29], the preoccupation subscale was not correlated with symptoms of depression. Thus, our findings suggest that mothers engage in bonding-related activities regardless of depressed mood and more or less regardless of the extent of EMSs. Bonding quality connects more strongly than quantity of bonding activities to the mood of the mother and her level of EMSs.

In theory [32], the EMS domain Disconnection and Rejection addresses negative assumptions regarding not having one’s emotional needs met by others, being different, distrusting others, pessimism, suppression of emotional expressions and fear of being exposed. Our results show that higher scores on this EMS domain are associated with a poorer quality of maternal-fetal bonding. This may be understood as a tendency to avoid emotional closeness with or to suppress warm feelings towards the fetus, thus affecting maternal bonding. The results correspond to research showing a relationship between several of the EMSs from the Disconnection and Rejection domain and the quality of interpersonal relationships (e.g., attachment style in adulthood; 43).

Overall, the results are in line with research supporting the link between EMSs and social relationships. The schema model is related to adult social functioning [44] and infant feeding difficulties [45, 46]. Earlier research has mainly looked to relationships in the past, suggesting that EMSs are developed from social experiences in childhood with important others [36, 37]. In contrast, we measured emerging relationships by exploring the mothers’ thoughts and feelings about their children before birth and the influence of infant temperament on the relationship.

The present study shed further light on the role of depressive symptoms in the associations between EMSs and maternal-fetal bonding. In line with previous research [48, 49], significant correlations between EMSs and depressive symptoms were found. The results of the mediation analyses showed indirect effects of the four EMS domains on bonding quality through depressive symptoms. However, significant direct effects of the EMS domains Disconnection and Rejection, Impaired Autonomy and Performance, and Impaired Limits were also found, suggesting that these EMS domains also affect maternal-fetal bonding independently of the effects of depression.

The results may have implications both for clinical practice and for research. Assessing EMSs may help clinicians identify pregnant women at risk for bonding difficulties. This may be important not only for preventing the development of a potentially unhealthy mother-child relationship but also for the treatment of women at risk. As this is the first study establishing a relationship between EMS domains and bonding quality, more studies are warranted. We encourage replication of our study as well as follow up studies with other measures of maternal-fetal bonding and with measures of bonding after birth. In addition, potential relationships between EMS domains and parent-infant interaction, infant attachment classification, and early child development should be explored. Preferably, the samples should include a higher proportion of disadvantaged families than the current sample. Clinical studies should investigate whether psychological interventions aimed at modifying EMSs can contribute to reducing bonding difficulties.

### Strengths and limitations

The longitudinal design of the study is a strength. The present study also has some limitations. First, only approximately 12% of all the pregnant women in the municipality of Tromsø were included in the study. The participation rate may partly be explained by failure to reach out to all pregnant women. In addition, the extensive data collection may have been perceived as demanding and time consuming by potential participants. Second, the present study sample consisted mostly of healthy and resourceful women. Although participants were recruited from a region with generally high socioeconomic status, their educational level and gross annual household income also indicate that the sample is quite resourceful. There is a possibility that a clinical or at-risk sample may have had less favorable scores on the study measures (MAAS, YSQ-S3, and EPDS) than our sample had. Due to the well-functioning sample, caution should be exercised in generalizing the findings to other populations. However, despite the resourcefulness and generally low levels of depressive symptoms in the study sample, it is worth mentioning that approximately one-third of participants reported having experienced depression in the past, possibly indicating some mental health vulnerability in the participants. Third, the predictors and maternal bonding were measured entirely by self-report questionnaires, which may have led to response bias. Measuring these variables with interviews may have given different results. Fourth, there were indications of possible multicollinearity for the EMS domains (although not at a level that raises serious concern), which may have affected the results of the hierarchical regression models. This means that the results from the hierarchical regression models should be interpreted with some caution. Fifth, given the large number of predictors in the regression analysis, an increased sample size would have been preferable.

## Conclusions

The present study has demonstrated that a mother’s EMSs are relevant to the quality of her bonding towards her fetus. After we controlled for confounding variables and the three other EMS domains, Disconnection and Rejection was a significant predictor of the quality of maternal-fetal bonding. Mothers’ symptoms of depression mediated the relationship between bonding quality and the EMS domains Disconnection and Rejection, Impaired Autonomy and Performance, Excessive Responsibility and Standards, and Impaired Limits. This is one of very few studies exploring cognitions and maternal-fetal bonding, and it is also the first study exploring the EMSs and maternal-fetal bonding. Our results are promising and call for more studies on cognition and bonding during pregnancy. In the future, assessing EMSs may enable improved identification of pregnant women at risk for bonding difficulties.

## Abbreviations

EMS: Early maladaptive schemas
EPDS: Edinburgh Postnatal Depression Scale
IPF: Intensity of preoccupation with the fetus
MAAS: Maternal Antenatal Attachment Scale
QMB: Quality of maternal bonding
YSQ: Young Schema Questionnaire
